# Hypergravity Activates a Pro-Angiogenic Homeostatic Response by Human Capillary Endothelial Cells

**DOI:** 10.3390/ijms21072354

**Published:** 2020-03-28

**Authors:** Chiara De Cesari, Ivana Barravecchia, Olga V. Pyankova, Matteo Vezza, Marco M. Germani, Francesca Scebba, Jack J. W. A. van Loon, Debora Angeloni

**Affiliations:** 1 Institute of Life Sciences, Scuola Superiore Sant’Anna, Via G. Moruzzi, 1, 56124 Pisa, Italy; decesari.chiara@gmail.com (C.D.C.); barravecchia.ivana@gmail.com (I.B.); olga.pyankova1407@gmail.com (O.V.P.); m.germani@sssup.it (M.M.G.); f.scebba@santannapisa.it (F.S.); 2 Department of Biology, University of Pisa, 1, 56124 Pisa, Italy; matteo.vezza@sns.it; 3 Department of Oral and Maxillofacial Surgery/Oral Pathology, Amsterdam UMC location VUmc and Academic Center for Dentistry Amsterdam (ACTA), Vrije Universiteit Amsterdam, Amsterdam 1081 HV, The Netherlands; j.vanloon@amsterdamumc.nl; 4 ESTEC, TEC-MMG-Lab, European Space Agency (ESA), Noordwijk 2201 AZ, The Netherlands

**Keywords:** angiogenesis, cytoskeleton, endothelium, F-actin, FAK, hypergravity, Large Diameter Centrifuge, vimentin, YAP1

## Abstract

Capillary endothelial cells are responsible for homeostatic responses to organismic and environmental stimulations. When malfunctioning, they may cause disease. Exposure to microgravity is known to have negative effects on astronauts’ physiology, the endothelium being a particularly sensitive organ. Microgravity-related dysfunctions are striking similar to the consequences of sedentary life, bed rest, and ageing on Earth. Among different countermeasures implemented to minimize the effects of microgravity, a promising one is artificial gravity. We examined the effects of hypergravity on human microvascular endothelial cells of dermal capillary origin (HMEC-1) treated at 4 *g* for 15 min, and at 20 *g* for 15 min, 3 and 6 h. We evaluated cell morphology, gene expression and 2D motility and function. We found a profound rearrangement of the cytoskeleton network, dose-dependent increase of Focal Adhesion kinase (FAK) phosphorylation and Yes-associated protein 1 (YAP1) expression, suggesting cell stiffening and increased proneness to motility. Transcriptome analysis showed expression changes of genes associated with cardiovascular homeostasis, nitric oxide production, angiogenesis, and inflammation. Hypergravity-treated cells also showed significantly improved motility and function (2D migration and tube formation). These results, expanding our knowledge about the homeostatic response of capillary endothelial cells, show that adaptation to hypergravity has opposite effect compared to microgravity on the same cell type.

## 1. Introduction

Since the 1960s, after the first orbital flight by Russian Cosmonaut Yuri Gagarin, humans have been spending extended periods of time aboard orbiting spacecrafts where the constant free-fall compensates the attraction of gravity, resulting in microgravity.

In space, microgravity induces an adaptive response that leads to a variety of symptoms because virtually all organs and organ systems are affected [1]. Vision problems caused by a combination of increased intraocular pressure and cosmic radiation have been also described [2]. Some of the reported symptoms are strikingly similar to the consequences of a sedentary life, senescence and degenerative diseases on Earth [3]. Indeed, prolonged best rest has been utilized as a model for the effects of microgravity [4]. 

A variety of countermeasures are implemented during and after space flight in the attempt to minimize the deleterious effects of microgravity. Astronauts receive dietary supplements, pharmacological treatment and above all, perform physical exercise. However, the most interesting countermeasure studied to date is probably artificial gravity [5], induced by means of centrifugation. Centrifugation has been tested against simulated and real microgravity in several human studies [6]. In-flight centrifuged crewmembers did not suffer from orthostatic intolerance and spatial disorientation; bed rest-induced cardiovascular deconditioning was also reduced [7]. Furthermore, daily exposure to hypergravity counteracted the muscular atrophy induced by prolonged bed rest [8]. Additionally, hypergravity treatment reduced muscle and bone atrophy also in rodents exposed to both real and simulated microgravity [9]. Exposure to hypergravity proved to be effective also in the treatment of common pathologies and ageing-related problems in different animal models. Chronic hypergravity showed a hormetic effect against allergic asthma and rhinitis, leading to hypothesize that using the centrifuge could alleviate allergic respiratory disorders [10]. Additionally, hypergravity was found effective in the stimulation of osteoblast production [11] and in neuroprotection against focal cerebral ischemia [12]. Hypergravity has also been proposed as an intervention to counteract obesity and some of the effects of aging in humans [13].

One of the tissues most affected by gravity changes is probably the endothelium, a diffuse organ constituted by the sum of all cells lining the inner surface of blood and lymphatic vessels. Mechanical stress applied to the surface of the cell is transferred through specific pathways to the nucleus and to adjacent cells [14], and converted into biochemical signals [15] that regulate basic homeostatic responses such as vascular tone, angiogenesis and inflammation [16,17]. Endothelial cells (ECs) adapting to microgravity change their proliferation rate, Nitric Oxide (NO) production, and cytoskeletal organization [18,19]. 

To gain a better basic understanding of the biological effect of gravitational load on a living system, it is necessary to test both reduction and increase of *g* values ergo to explore the gravity continuum [20]. For example, short-term application of hypergravity improves the endothelial barrier integrity [21]. 

In this work, we examined potential mechanisms underlying the effects of hypergravity. Our previous work demonstrated changes in the morphology and gene transcription of human microvascular endothelial cells of dermal origin (HMEC-1, [22]), the same cell type that we sent to space flight [23]. In the current work reported here cells were exposed to 4 *g*, a low level of stimulation close to a feasible, short-period human treatment, and to 20 *g*, a very high level that we used to study the system reaction to an extreme stimulation. Experiments were performed at the Large Diameter Centrifuge (LDC) facility of the European Space Research and Technology Centre (ESTEC)—European Space Agency (ESA) (Noordwijk, The Netherlands), within the 2016 “Spin Your Thesis” activities. 

We found that exposure to hypergravity affects the morphology, transcriptome and function of capillary EC, suggesting overall that hypergravity has opposing effects compared to microgravity on capillary ECs.

## 2. Results

**Figure 1:**
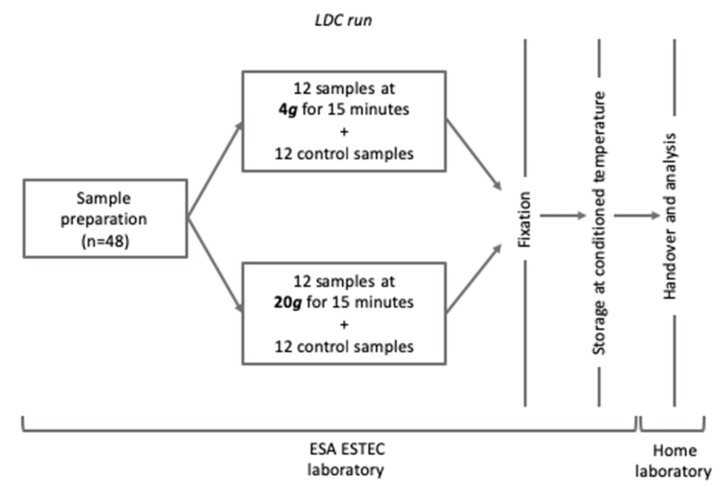
Workflow of the experimental treatment at the Large Diameter Centrifuge (LDC) facility (Noordwijk, NL). After runs, all samples were fixed and prepared for transportation to the home laboratory for endpoint analysis.

HMEC-1 cells were exposed to hypergravity both using the LDC (4 *g* or 20 *g* for 15 min), within the Spin Your Thesis 2016 campaign at ESA-ESTEC (Noordwijk, NL), Figure 1, and ordinary centrifuges at the home laboratory (20 *g*, for 1, 3 and 6 h). The samples were fixed with NOTOXhisto (Scientific Device Laboratory, Des Plaines, IL, USA) for morphological analysis and with RNAprotect (Qiagen, Germantown, MD, USA) for transcriptome analysis. 

### 2.1. Effects of Short Hypergravity Treatments

The cytoskeleton is deeply affected by changes of gravitational load. For this reason, we choose to analyze different cytoskeletal markers and adhesion molecules. 24 samples were exposed to either 4 *g* or 20 *g* for 15 min and then fixed and stained. 

**Figure 2:**
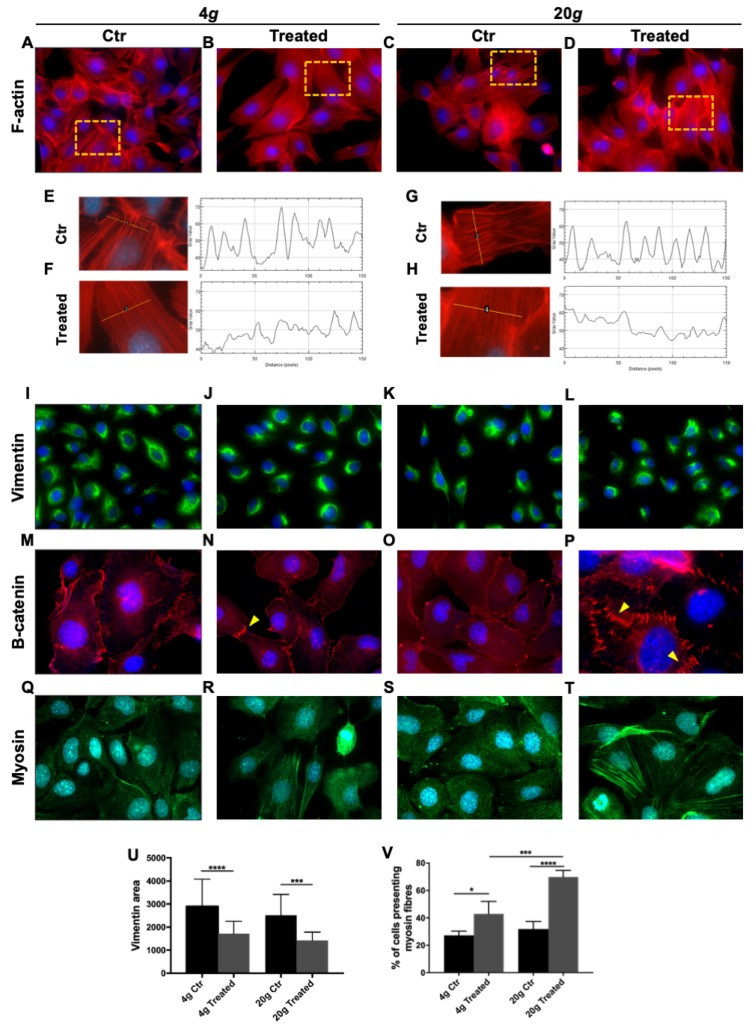
Effect of short hypergravity treatments. Human microvascular endothelial cells HMEC-1, of dermal origin, exposed to 4 *g* or 20 *g* in the Large Diameter Centrifuge (LDC) and respective controls. (**A–D**) Red: F-actin; magnification: 63×. (**E–H**) The analysis of F-actin fluorescence intensity profile was performed with Plot Profile plug of ImageJ suite on several representative cells. In treated cells the plot profile showed an increased number of peaks but a decrease of their intensity. (**I–L**) Green: vimentin; magnification: 63×. (**M–P**) Red: catenin-beta; yellow arrowheads mark cell junctions; magnification m,n,o: 63×; magnification p: 100×. (**Q–T**) Green: myosin; magnification: 100×. Nuclei are stained in blue (4′,6-diamidino-2-phenylindole, DAPI). (**U**) Measurement of the area (pixel) stained for vimentin showed a statistically significant more compact distribution of the marker in hypergravity-treated cells compared with respective controls. Although the effect seems stronger after 20 *g*, it is not statistically different from 4 *g*. (**V**) Myosin is represented by detectable fibers in a higher number of cells in samples treated with hypergravity. The effect is dose dependent. All images were analyzed with ImageJ software. All data are means ± SEM. We analyzed at least 20 cells per experimental group. All experiments were repeated at least three times, each time with three replicas; One-way Anova test, * *p* < 0.05, *** *p* < 0.001, **** *p* < 0.0001.

F-actin staining showed thick stress fibers in control samples, while hypergravity-treated cells showed thinner but much denser fibers (Figure 2A–D). F-actin fluorescence intensity profile was inversely dose-dependent, the higher the level of hypergravity the lower the peak amplitude (Figure 2E–H). 

Vimentin distribution was also affected by exposure to hypergravity. Control cells featured the usual perinuclear spreading of the protein, whereas treated cells showed a much more compact distribution around nuclei. One-way Anova test showed a significant difference between control and treated samples at each value of hypergravity tested, but not any significant difference between 4 and 20 *g* (Figure 2I–L, U).

Once we verified that two important cytoskeleton components as microfilaments (F-actin) and intermediate filaments, IF, (vimentin is a representative of type III IF) were affected even by a short period of hypergravity, we went on to study the distribution of catenin-beta at cell adherens junctions. While in control samples catenin-beta staining was more intensely cytoplasmic, in treated samples it localized at the cell borders forming interdigitated and belt-like junction zones between cells (Figure 2M–P, yellow arrowheads). The effect was somewhat more pronounced at 20 *g* compared to 4 *g*.

We then investigated the effect of hypergravity on indicators of cell motility. We stained a set of samples for myosin and observed two different modalities of organization, either diffused in the cytoplasm or organized in fibers. In treated samples, the percentage of cells that presented detectable fibers was significantly higher compared to control, with samples exposed to 20 *g* showing a higher percentage in comparison to cells at 4 *g* (Figure 2Q–T,V).

To determine whether a short hypergravity stimulation could affect gene transcription, we performed a transcriptome analysis on samples fixed with RNAprotect using RT^2^ profiler PCR arrays, specific for ECs (CFX96 Touch Real-Time PCR Detection System, BioRad, Hercules, CA, USA). 

The results showed up-regulation of genes promoting nitric oxide (NO) production, endothelial activation and angiogenesis, both after 4 *g* and 20 *g* treatments (Table 1). Also, at 20 *g* we observed a down-regulation of Thrombomodulin (*THBD*). Interestingly, Von Willebrand Factor (*VWF*) showed down-regulation after 4 *g* and up-regulation after 20 *g* treatment. 

To summarize, the transcriptome analysis showed variation in the expression of genes associated with cardiovascular homeostasis, NO production, angiogenesis, and inflammation. 

### 2.2. Effect of Longer Hypergravity Treatments

**Figure 3:**
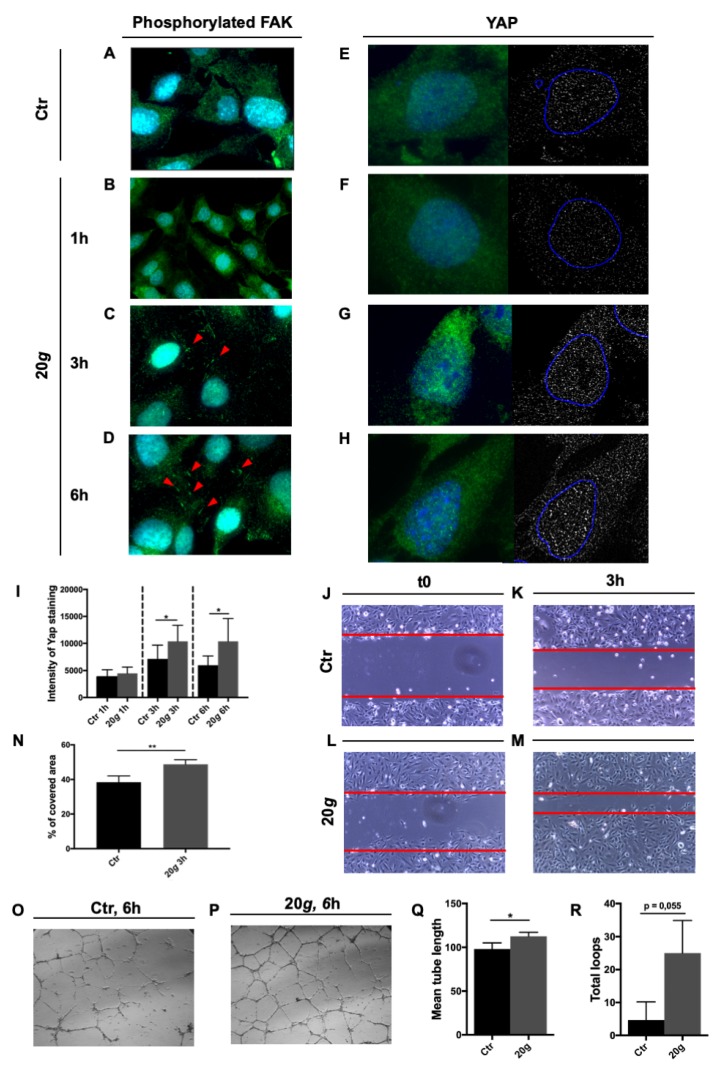
Effect of long hypergravity treatments. Human microvascular endothelial cells HMEC-1 exposed to 20 *g* in the short radius 5804 Eppendorf centrifuge and respective controls. (**A–D**) Green (red arrowheads): Phosphorylated Focal adhesion kinase (FAK); magnification (**A**,**C**,**D**): 100×; magnification b: 63×. (**E–H**) Yes-associated protein 1 (YAP1) immunofluorescence signal in green on the left and in white on the right; blue circle on the right: nuclei perimeter. Magnification: 100×. Images were analyzed with ImageJ software. Nuclei are stained in blue (4′,6-diamidino-2-phenylindole, DAPI). (**I**) YAP1 staining intensity showed a statistically significant increase in hypergravity treated cells compared with reference (unit: pixel). The effect is detectable both after 3 and 6h treatment. (**J–M**) Representative results of 3 h wound healing assay. Magnification: 10×. (**N**) Percentage of covered area in a wound healing assay after 3 h of hypergravity treatment is higher in treated cells than in control. (**O–P**) Representative results of a 6 h tube formation assay on Geltrex (Thermo Fisher Scientific, Waltham, MA, USA). Magnification: 5×. (**Q–R**) Mean tube length (unit: pixel) is higher in treated cells than in control. The total loop number, also, shows an increasing trend in treated cells. All data are means ± SEM. All experiments were repeated at least three times, each time with three replicas; Student’s t-test for unpaired samples, * *p* < 0.05, ** *p* < 0.01.

Following the results obtained with short hypergravity treatments we speculated that the system could be stressed further, with a longer treatment, especially because highly complex structures like focal adhesions (FA) might necessitate more time to make modifications evident. Cells exposed to hypergravity showed increased, dose-dependent tyrosine-phosphorylation of Focal Adhesion kinase (FAK), undetectable at T0 and after 1 h (Figure 3A–B), but increasingly more evident from 3 to 6 h (Figure 3C–D).

Yes-associated protein 1 (YAP1) acts as a mechanosensor involved in maintaining tissue tension and shape and in cellular motility and migration [24]. We found an increase of YAP1 protein expression following hypergravity treatment. After 1 h there was not any difference among treated and reference cells (Figure 3E–F), whereas a significant increase of protein staining was visible both after 3 and 6 h (Figure 3G–H,I).

Two basic functional assays (wound healing and tube formation) were performed to assess the effect of hypergravity on HMEC-1 function. We found that the molecular events registered (i.e., modification of myosin organization, increased FAK phosphorylation, and increased YAP1 expression) were associated with a significant increase of 2D migration by HMEC-1 at 3 h, 20 *g* hypergravity compared to reference cells (Figure 3J–N). Additionally, a 6-h hypergravity treatment at 20 *g* induced a significant increase of mean tube length along with a positive trend (although not statistically significant) toward increase of the number of loops (Figure 3O–R).

## 3. Discussion

In this work we explored the effect of hypergravity as a possible countermeasure to extensive adaptations to microgravity enacted by ECs, studying the same cell type that we had previously sent to the International Space Station (ISS) [18].

We observed a diametrical difference between the effects of hypergravity and microgravity on F-actin fibers. Hypergravity, in a dose-dependent effect, produced more numerous and thinner F-actin fibers (Figure 2A–H), strikingly different from the F-actin organization observed in the same cell type after culture in space [23]. Actin remodeling is particularly interesting in that it underlies a large number of basilar cell phenomena. In ECs, stabilization of actin polymerization is required for NO production through direct effect on arginine transport [25]. Actin microfilaments are peculiarly susceptible to gravitational variations, suggesting that the cytoskeleton is the cell gravisensor [26]. Similar but not identical reorganization of actin was reported in other EC models, variations probably due to different experimental hypergravity treatments: Bovine Aortic Endothelial Cells (BAEC) showed an increase of stress fibers in the cytoplasm and loss of peripheral rings after 5 periods of 10 min exposure to 10 *g* alternated with 10 min at 1 *g* [27], and a similar response after 3 min exposure to 3 *g*, in addition to inhibition of stress fibers formation after 10 min [28]; Human Umbilical Vein Endothelial Cells (HUVEC) featured a significant enforcement of peripheral F-actin after exposure to 2 *g* for 15 min [21]. In general, hypergravity induced an increase in number of stress fibers or an expansion of their diameter.

Gene expression analysis of HMEC-1 treated with 20 *g* for 15 min showed a significant up-regulation of *NOS3*, the enzyme primarily responsible for the generation of NO in the vascular endothelium [29]. NO plays a key role to maintain quiescence in the vascular wall by inhibiting cell proliferation, inflammation and thrombosis. NO mediates vascular endothelial growth factor (VEGF)-induced angiogenesis in coronary vessels [29] and it is considered a potential tool for local therapy in vascular disease [30]. ECs modulate NO production in response to environmental stress including gravitational load, although the response is not homogeneous through different systems. In fact, NO increase was reported both in microgravity- [19] and hypergravity-treated HUVEC (a model of macrovascular ECs) [31], while another endothelial-related cell line, EA.hy926, showed down-regulation of *NOS3* after hypergravity [32]. 

Other genes associated with enhancement of NO production, namely *APOE* and *IL1beta*, were found up-regulated after 15 min at 20 *g*. *IL1beta* is an important mediator of the inflammatory response, involved in controlling cell proliferation and differentiation. During inflammation, it can increase permeability of endothelium [33]. Moreover, *IL1beta* induces NO generation in vascular endothelium by up-regulating *NOS* genes expression [34,35]. *APOE* participates to transportation of lipoproteins, cholesterol and fat-soluble vitamins and it is also involved in NO production [36]. 

The gene expression scenario we found describes a general increase in NO production though different pathways. 

We found oher interleukines, in addition to *IL1beta*, up-regulated upon hypergravity: *TNFSF10* and *FASLG*. These two ligands are involved in the regulation of the inflammatory response and the modulation of the immune system [37]. Interestingly, *TNFSF10* also induces a selective apoptosis in a variety of transformed and tumor cells, but not in most normal cells [38], so much so, that several clinical trials are testing its use against different types of tumor [39,40].

Another vasorelaxant hormone that we found up-regulated after exposure to 4 *g* for 15 min is *NPPB*, a member of the natriuretic peptide family. *NPPB* encodes for a secreted protein involved in natriuresis, diuresis, vasorelaxation, inhibition of renin and aldosterone secretion, thus playing a key role in cardiovascular homeostasis [41].

Actin colocalizes with myosin in EC cytoskeleton. Their interaction is essential to regulate the width of intercellular clefts, thereby controlling vascular permeability [42] which is crucial in maintaining circulatory homeostasis and organ function. A dose dependent increase of both actin stress fibers and myosin fibers was observed in HMEC-1 after hypergravity (Figure 2Q–T,V). Myosin is essential for cell movements [43] and that could explain why hypergravity significantly increased HMEC-1 performance in the wound healing assay (Figure 3). These is consistent with an increase of migration rate observed after hypergravity treatment also in other ECs such as HUVEC and BAEC [28].

The increase in migration rate might be further stimulated by the increased phosphorylated FAK, observed in hypergravity treated HMEC-1 (Figure 3A–C) not before 3 h but with a strong dose-dependent effect for up to 6 h. The phosphorylation of FAK is triggered by integrin-mediated cell adhesion [44] and its activity has a critical role in angiogenesis and cell migration [45,46]. 

Hypergravity also modified the IF compartment causing tight accumulation of vimentin around the nucleus (Figure 2I–L), the opposite of what we observed in space flown HMEC-1 cells, where vimentin showed well-defined fibers, distributed all through the cytoplasm (Barravecchia et al., personal communication). The redistribution was not significantly dissimilar between 4 and 20 *g*, suggesting that the lower treatment was enough to reach a plateau. Interestingly, a formation of dense vimentin aggregates was observed in other cell lines after exposure to simulated microgravity: papillary thyroid carcinoma cells (exposed to clinostat for 30 min [47]), human chondrocytes (Random Positioning Machines, (RPM) for 24 h [48]), breast cancer cells (RPM for 24 h [49]). Therefore, reorganization of vimentin, that is involved in cell adhesion, motility, and in maintaining nuclear shape [50], may represent an adaptive mechanism to stress [51] that takes on different forms in different cell models. 

Endothelial adherens junctions play a central role in maintaining vascular permeability [52]. In this context, catenin-beta provides stability to the cell monolayer under stress [53] and mediates adhesive functions. To our knowledge, we were the first to analyze catenin-beta distribution in hypergravity-treated ECs, and the reorganization we observed in HMEC-1 could represent an adaptive mechanism to protect cells from outer forces and to strengthen tissue integrity and impermeability. 

YAP1 is a transcription factor of the Hippo signaling pathway. In addition to being involved in cell proliferation and death [54], YAP1 is a mechano-sensor [24,55] and its activation is required for mechanical strain-induced cell-cycle re-entry [56]. Increase of F-actin polymerization is associated to an increase in YAP1 activity [57]. The significant up-regulation of YAP1 protein and cytoskeletal adaptations we found, suggest a general stiffening of hypergravity-treated cells, which occurs between 1 and 3 h of treatment, and is coherent with increase of cell motility (see functional tests, Figure 3) but also with other recent LDC results [58] showing increased cell membranes viscosity in osteoblastic and endothelial cells exposed to 15 *g*.

Based on the literature [59,60,61,62], we expected gene expression changes associated with apoptosis. Indeed, although the experimental treatment was brief, *CFLAR*, a factor that protects against apoptosis [63], was found up-regulated in HMEC-1 cells upon hypergravity, our result being coherent with data from other cell types [27,64,65].

*MMP1* (Collagenase-1) and *MMP2* (Gelatinase-A) genes were also significantly up-regulated by hypergravity. Both are members of matrix metalloproteinases (MMPs) family and are pro-angiogenic factors, involved in the breakdown of extracellular matrix in normal physiological processes such as embryonic development, reproduction, and tissue remodeling [66]. In vitro, endothelial tube formation is blocked by MMPs inhibitors [67] and it is increased by recombinant MMP2 [68]. A third gene involved in angiogenesis, *PLG*, was found up-regulated after 4 *g*. PLG protein is activated by proteolysis and converted to plasmin. The PLG/plasmin system is involved in the degradation of extracellular matrix during cell migration, tissue remodeling, wound healing, angiogenesis and inflammation [69]. All the above is coherent with the striking results of the tube formation assay after exposure to 20 *g* (Figure 3).

A most interesting result obtained from the transcriptome analysis regarded the expression of *VWF*. The gene was found down-regulated after 4 *g* and up-regulated after 20 *g*. VWF is a plasma glycoprotein involved in physiological platelet tethering. Its interaction with GPIbα mediates platelet adhesion [70] and for this reason it contributes to thrombotic disorders following endothelial and platelet dysfunction [71,72]. *VWF* deregulation is increasingly correlated with a higher incidence of heart attack and stroke [73,74]. High plasma levels of VWF correlated with an increased risk of thrombotic diseases [75]. On these bases, the control of *VWF* expression has been proposed as an interesting therapeutic tool to treat thrombotic diseases such a stroke and myocardial infarction [76]. Our results may support this hypothesis. In fact, a short 4 *g* treatment was sufficient to reduce the expression of *VWF*. Possible therapeutic hypergravity treatments could range between 2 and 2.5 *g*, values that are close to the lower level used in our experiment, so that it would be interesting to evaluate *VWF* expression changes at 2.5 *g*.

**Figure 4:**
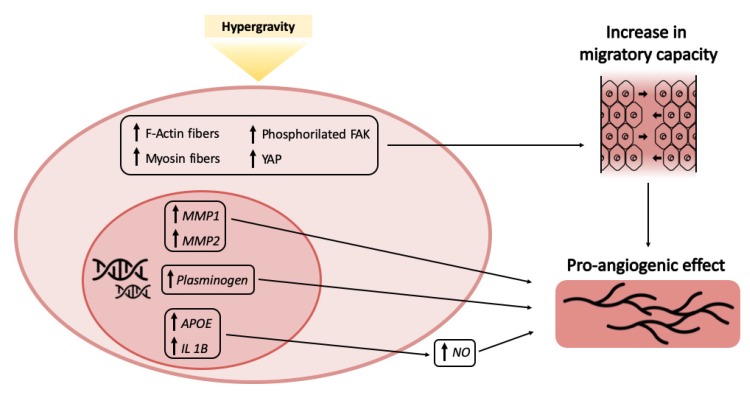
Modeling of concurring events resulting in the pro-angiogenic effect of hypergravity.

In summary, the results presented here are a preliminary indication that hypergravity treatments elicit in capillary ECs a complex response suggesting increase in cell motility and stimulation of angiogenesis at large (Figure 4).

Different levels and duration of hypergravity treatments should be further tested to better evaluate this possible positive effect on cardiovascular homeostasis, angiogenesis and inflammatory response.

A direct comparison is not possible due to the experimental logistics, however from a qualitative point of view the human capillary ECs evaluated in our studies offer a response to hypergravity that is opposite in sign to microgravity.

## 4. Materials and Methods 

### 4.1. Cell line and Culture

HMEC-1 (Human Microvascular Endothelial Cells, CDC, Atlanta, GA, USA) is an immortalized EC line derived from human dermal capillaries [21]. Cells, cultured as in [17], were kept in MCDB-131 medium supplemented with 1µg/mL Hydrocortisone, 10ng/mL Epidermal Growth Factor (EGF), 1mM Sodium bicarbonate, 10% FBS (Thermo Fisher Scientific, Waltham, MA, USA), 1U/mL–1µg/mL Penicillin/Streptomycin, on Cyclic Olefin Copolymer (COC, IBIDI, Martinsried, Germany) coverslips, chosen for their good resistance to mechanical stress, and optical transparency for subsequent fluorescence microscopy. To improve adhesion, flasks and coverslips were coated with 2% pork skin gelatine, 20 min at Room Temperature (RT). 

Hypergravity treatments were performed at ESTEC, with 90% confluent monolayers of HMEC-1 cells seeded on COC coverslips inserted in 6-well plates, containing 2mL cell culture medium. Samples were fixed directly within the 6-well plates, in equal number for either nucleic acid extraction or for immunofluorescence analysis. For robust statistics, three biological replicas were used for both sample types. 

Fixed samples were brought back to home lab in the appropriate storing solutions: 1×PBS for the samples fixed with NOTOXhisto (Scientific Device Laboratory, Des Plaines, IL, USA) and prepared for immunofluorescence, and RNAProtect Cell Reagent (Qiagen, Germantown, MD, USA) 1:6 in 1× PBS for RNA extraction. All samples were transported at 4 °C.

### 4.2. Reagents

All reagents, growth media and supplements were from Sigma-Aldrich unless specified differently.

### 4.3. Hypergravity Protocols

Short-duration treatments were performed at the Large Diameter Centrifuge (LDC) laboratory of the ESA-ESTEC facility, Noordwijk (NL). The LDC has a diameter of 8 m and four arms that hold at maximum six gondolas, plus one central gondola. Gondolas swing out, resulting in an acceleration vector that is always perpendicular to the sample surface [77]. The large diameter reduced the impact of inertial shear related to rotation considerably [78]. Cells were exposed to two levels of mechanical loading, 4 *g* and 20 *g*, for 15 min each. Experimental samples were hosted in the swing out gondolas while reference samples were kept at 1 *g* inside the central gondola, to include the influence of residual vibration into the analysis.

In addition, although short-radius centrifuges commonly used in laboratory settings cannot avoid some inertial shear stress, for qualitative analysis we performed longer treatments in the home laboratory. We used the 5804 Eppendorf centrifuge, taking advantage of the small sample size and the swing out rotor (A-2-DWP) configuration, exposing cells to 20 *g* for 1, 3 and 6 h.

### 4.4. Wound Healing (Scratch) Assay

HMEC-1 cells (200000) were seeded in 12-well plates to yield confluent monolayers in 24 h. Cells were then exposed to 20 *g* for 3 h. Afterwards, scratches were generated with sterile 200µL pipette tip. Cells were then incubated in a standard cell culture incubator at 37°C (5% CO_2_) for 5 h.

Images were acquired with Leica DM IL LED microscope, 10× objective, at time of scratch (T0) and after 5 h (T5). The extent of cell migration was measured with ImageJ 1.50i software. The migratory rate was determined as a relative percentage of scratch closure at T5 compared to T0.

### 4.5. Tube Formation Assay

HMEC-1 cells (15000 per well) were seeded in IBIDI micro-slides angiogenesis plates previously coated with Geltrex (37°C for 30 min). Cells were then incubated in a standard cell culture incubator at 37°C (5% CO_2_) for 1 h to allow the attachment, and subsequently exposed to with 20 *g* for 5 h. Images were acquired with Leica DM IL LED microscope, 10× objective, 24 h after seeding. The extent of tube formation was measured with Wimasis image analysis software.

### 4.6. Antibodies, Cell Staining and Fluorescence Analysis 

The following primary antibodies were used: catenin-beta (Thermo Fisher Scientific, Waltham, MA, USA): 1:100; vimentin: 1:200; myosin light chain 2 (Cell Signaling, Danvers, MA, USA): 1:200; FAK [pY861] (Invitrogen, Carlsbad, CA, USA): 1:200; YAP1 (Santa Cruz Biotechnology, Dallas, TX, USA): 1:200. Secondary antibodies: Alexa Fluor 568 donkey anti-mouse IgG, Alexa Fluor 488 donkey anti-mouse IgG, Alexa Fluor 488 donkey anti-rabbit IgG, 1:500 (Molecular Probes, Eugene, OR, USA).

Cells prepared for immunocytochemistry were first washed in NOTOXhisto (Scientific Device Laboratory, Des Plaines, IL, USA) 1:2 in 1× PBS, then fixed in NOTOXhisto 1:2 in 1× PBS for 30 min and stored in 1× PBS at 6°C until use. Cells were then washed in 1× PBS, permeabilized with 0,1% Tryton in 1× PBS (4 min at RT), incubated in blocking solution (1% Bovine Serum Albumin, BSA, in 1× PBS-Tween 0,1%), 30 min at RT, exposed to the primary antibody in blocking solution (1 h at 37 °C), washed again with 1× PBS-Tween 0,1% and incubated with the secondary antibody and/or Phalloidin staining (Act 550-phalloidin, 1:200) in blocking solution (1 h at RT). Cells were washed again with 1× PBS-Tween 0,1% and stained with 4′,6-diamidino-2-phenylindole (DAPI, 1:1000 in 1× PBS, 10 min at RT). Finally, samples were rinsed twice with 1× PBS and mounted with Aqua Poly-Mount (Polysciences, Warrington, PA, USA). 

Images were acquired with Axioscope 40 and PALM MicroBeam Microscope (Zeiss, Oberkochen, Germany). Pictures analysis was performed with ImageJ 1.50i software.

### 4.7. Cell Fixation and RNA Extraction

Cells prepared for nucleic acids extraction were washed with 1× PBS and fixed with RNAprotect Cell Reagent (Qiagen, Germantown, MD, USA) 1:6 in 1× PBS for 30 min, twice. Samples were then stored in this solution at 6°C, until use. Total RNA was isolated with ReliaPrep RNA Cell Miniprep System (Promega, Madison, WI, USA), quantified spectrophotometrically (NanoDrop ND-1000, NanoDrop Technologies, Wilmington, DE, USA), and quality-checked (Bioanalyzer 2100, Agilent Technologies, Santa Clara, CA, USA). Sample quality and integrity were visually evaluated with 2% agarose gel electrophoresis.

### 4.8. RT^2^ Profiler PCR Arrays

Transcriptome analysis was performed with RT^2^ Profiler PCR Arrays (96-well format) for Human Endothelial Cell Biology (Qiagen, Germantown, MD, USA). 100 ng total RNA was used to generate cDNA using the RT^2^ First Strand Kit (Qiagen, Germantown, MD, USA); qRT-PCR was performed with CFX96 Touch Real-Time PCR Detection System (BioRad, Hercules, CA, USA). Relative expression was determined with the -∆∆CT method. 

### 4.9. Statistical Analysis

Data of immunofluorescence were obtained from at least three independent experiments. All data are indicated as mean±SEM and were analyzed with Student’s t-test for unpaired samples. Statistical analysis was performed with GraphPad software. Difference between means was judged statistically significant for p-value ≤ 0.05. Data from gene expression analysis were obtained from three independent experiments (each performed with triplicates) and were analyzed at www.SABiosciences.com/pcrarraydataanalysis.php. Reference genes were provided by the system (*GAPDH*, *RPL0*, *ACTB*, *B2M*, *HPRT1*) as well as internal technical controls (see Manufacturer’s manuals). 

## Figures and Tables

**Table 1:** Up- or down-regulated genes in the listed comparisons, after 15 min exposure to either 4 *g* or 20 *g* in the Large Diameter Centrifuge (LDC). Only genes with *p* < 0.05 were reported.

	4*g* Vs. Ctr	20*g* Vs. Ctr	20*g* Vs. 4g
**Up**	*FASLG, NPPB, PLG.*	*TNFSF10, IL1β, ICAM1, TGFB1, MMP2, CFLAR, APOE, COL18A1, FN1, NOS3, PLAU, VWF.*	*ENG, MMP1, PLAT, PROCR, SERPINE1, TEK, TFPI, TGFB1, VWF, ACTB, GAPDH, NOS3.*
**Down**	*VWF*	*THBD*	

*FASLG*: Fas Ligand. *NPPB*: Natriuretic Peptide B. *PLG*: Plasminogen. *VWF*: Von Willebrand Factor. *TNFSF10*: TNF Superfamily Member 10. *IL1β*: Interleukin 1 beta. *ICAM1*: Intercellular Adhesion Molecule 1. *TGFB1*: Transforming Growth Factor Beta 1. *MMP2*: Matrix Metallopeptidase 2. *CFLAR*: CASP8 and FADD Like Apoptosis Regulator. *APOE*: Apolipoprotein E. *COL18A1*: Collagen Type XVIII Alpha 1 Chain. *FN1*: Fibronectin 1. *NOS3*: Nitric Oxide Synthase 3. *PLAU*: Plasminogen Activator Urokinase. *THBD*: Thrombomodulin. *ENG*: Endoglin. *MMP1*: Matrix Metallopeptidase 1. *PLAT*: Plasminogen Activator, Tissue Type. *PROCR*: Protein C Receptor. *SERPINE1*: Serpin Family E Member 1. *TEK*: TEK Receptor Tyrosine Kinase. *TFPI*: Tissue Factor Pathway Inhibitor. *ACTB*: Actin Beta. *GAPDH*: Glyceraldehyde-3-Phosphate Dehydrogenase.

## Supplementary Materials

Supplementary File 1

## Abbreviations

Anova	Analysis of Variance
B2M	Beta-2-Microglobulin
BAEC	Bovine Aortic Endothelial Cells
DAPI	4′,6-diamidino-2-phenylindole
EC	Endothelial Cells
ESA	European Space Agency
ESTEC	European Space Research and Technology Centre
FAK	Focal Adhesion Kinase
HMEC-1	human microvascular endothelial cells 1
HPRT1	Hypoxanthine Phosphoribosyltransferase 1
HUVEC	human umbilical vein endothelial cells
ICAM1	Intercellular Adhesion Molecule 1
IF	intermediate filaments
ISS	International Space Station
LDC	Large Diameter Centrifuge
NO	Nitric Oxide
NOS	Nitric Oxide Synthase
PCR	Polymerase Chain Reaction
PROCR	Protein C Receptor
RPL0	Ribosomal Protein Lateral Stalk Subunit P0
RPM	Random Positioning Machines
RT	room temperature
SANS	space flight-associated neuro-ocular syndrome
SEM	standard error of the mean
SYT	Spin Your Thesis
YAP	Yes-Associated Protein 1
