# Supplementary material for: Hypergravity Activates a Pro-Angiogenic Homeostatic Response by Human Capillary Endothelial Cells
Source: Int J Mol Sci. 2020 Mar 28;21(7):2354. doi: 10.3390/ijms21072354 (PMC7177524; doi:10.3390/ijms21072354)
Supplement: Supplementary File 1 [file ijms-21-02354-s001.pdf]

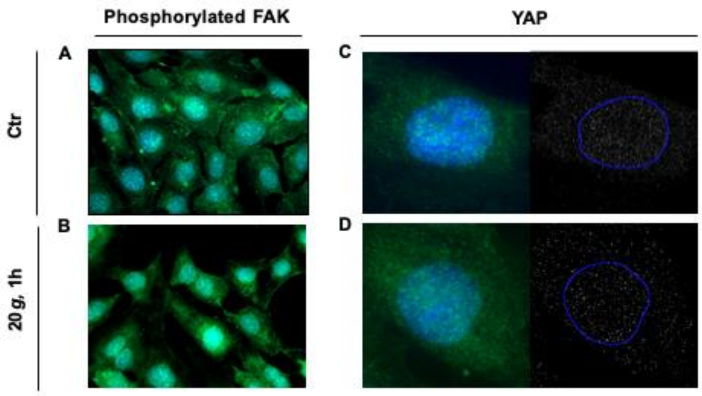

Supplementary Figure 1. HMEC-1 exposed to 20 *g* for 1 hour and respective controls.

(a-b) Green: Phosphorylated FAK; magnification: 100X. (c-d) YAP1 immunofluorescence signal in green on the left and in white on the right; blue circle on the right: nuclei perimeter. Magnification: 100X. Images were analyzed with ImageJ software. Nuclei are stained in blue (DAPI). 1 hour 20 *g* hypergravity treatment does not cause detectable changes of the aforementioned markers in HMEC1 cells.
